# Natural Product Type III Secretion System Inhibitors

**DOI:** 10.3390/antibiotics8040162

**Published:** 2019-09-24

**Authors:** Heather A. Pendergrass, Aaron E. May

**Affiliations:** Department of Medicinal Chemistry, Virginia Commonwealth University, Richmond, VA 23284, USA; pendergrassha@vcu.edu

**Keywords:** natural products, pathogenesis, type III secretion system, probiotics, prophylaxis

## Abstract

Many known inhibitors of the bacterial type III secretion system (T3SS), a virulence factor used by pathogenic bacteria to infect host cells, are natural products. These compounds, produced by bacteria, fungi, and plants, may have developed as prophylactic treatments for potential attack by bacterial pathogens or as an attempt by symbiotic organisms to protect their hosts. Regardless, better understanding of the structures and mechanisms of action of these compounds may open opportunities for drug development against diseases caused by pathogens utilizing the T3SS. This review will cover selected known natural products of the T3SS and detail what is known of their origin and mechanism of action. These inhibitors highlight nature’s ability to modulate interactions between organisms at a cellular level.

## 1. Introduction

The type III secretion system (T3SS) is a virulence factor utilized by many Gram-negative pathogens to enable and perpetuate infection of a host [1,2,3]. Pathogens known to encode a T3SS include enteropathogenic and enterohemorrhagic *Escherichia coli* (EPEC and EHEC, respectively) [4,5,6], *Salmonella enterica* serovar Typhimurium [1], *Chlamydia* species [7], *Yersinia pestis* [2,8,9], *Vibrio* spp., *Shigella* spp., and *Pseudomonas* spp. [2]. The T3SS is indispensable in the ability of a pathogen to cause infection, with knockouts of the T3SS being avirulent [10]. Chemical inhibition of the T3SS has emerged as a strategy to combat these pathogens [11]. Inhibition of the T3SS results in an inability of a pathogen to cause infection in a host; in vivo studies in mice have shown that T3SS inhibitors allowed the host immune system to clear the infection better than a placebo [12,13,14]. In addition, a functioning T3SS is not necessary for bacterial cell viability, and inhibition of the T3SS is not toxic to the pathogen [15]. This removes the selective pressure for the formation of resistance to treatment [16].

**Figure 1:**
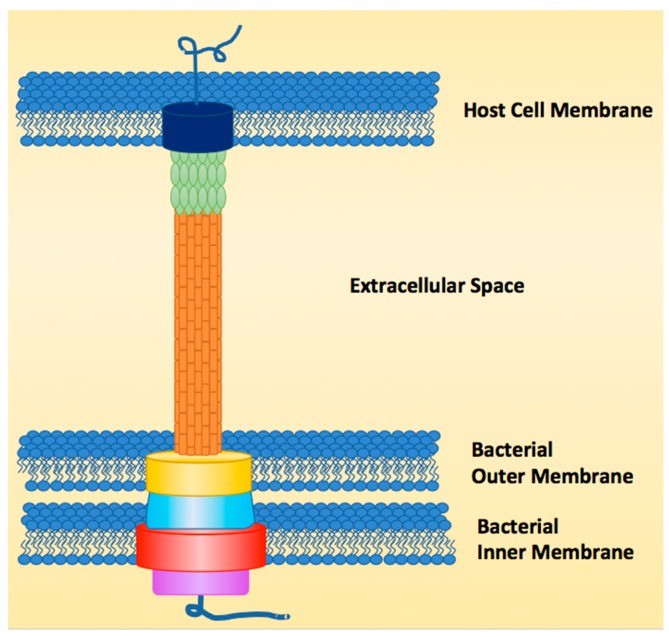
Example of type III secretion system (T3SS) structure.

The T3SS functions like a molecular syringe by injecting or secreting effector proteins directly from the cytosol of the bacterial pathogen through the host cell membrane, earning it the nickname “the type III injectosome” [2,4,17,18,19,20]. The structure of the injectosome can be broken down into three major regions, the sorting platform, the basal body and the needle (Figure 1). The sorting platform, which is within the cytoplasmic region of the bacterial cell, contains an ATPase (purple) to power secretion of linear unfolded proteins, as folded proteins are too wide to go through the ~2.5 nm needle [21,22]. The ATPase also functions as the recognition domain for effectors and unfolds the effectors for secretion. The basal body (yellow, blue, red) is made of a dual ring system that spans the inner and outer bacterial membranes and anchors the needle to the bacterial cell surface. The needle is composed of helical monomers (orange) that form a tube-like structure after polymerization [20]. The needle differs in length from species to strain depending on the host that the pathogen has evolved to infect [23]. The tip of the needle (green) also varies in length from one species to another. In *E. coli*, for instance, the needle tip is longer than the needle itself, which is not the case for other T3SSs [20]. The final structural component to the injectosome is the pore (dark blue) that is formed in the host cell membrane, made up of two pore-forming proteins that allow effectors to pass into the host (for *E. coli*, EspB and D) [24,25,26,27]. 

Once the effectors pass into the host cell, they elicit specific responses from the host. In the case of *E. coli*, one such effector is translocated intimin receptor (Tir) [3,10,28,29,30]. Tir is secreted in an unfolded form into the host cell. Once there, it folds and presents itself on the surface of the host cell. Intimin, which is presented on the outer membrane of *E. coli*, recognizes the translocated Tir and binds. This forms an intimate attachment between the bacteria and the host cell. This attachment is necessary for the propagation of infection [26]. Other activities elicited by effector proteins include mimicking host signaling proteins and enzymes to hijack host cell machinery, inducing host cell death directly, or evading immune response in the host cell. Up to 30 different effector proteins can be secreted by a single pathogen [31,32,33].

**Figure 2:**
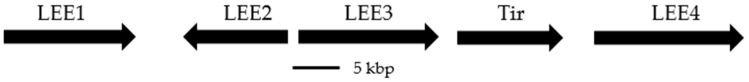
Gene map for the locus of enterocyte effacement (LEE) in *E. coli*, indicating the five major promoter regions [40].

The T3SS and its components are typically encoded in pathogenicity islands (e.g., *Salmonella* pathogenicity islands, SPIs [34,35,36]); however, in the case of *Yersinia* species, the genes for the T3SS can also be found on a ~70 kbp virulence plasmid [8,37]. The ~35 kbp pathogenicity island that encodes the T3SS in *E. coli* is referred to as the locus of enterocyte effacement (LEE) [5,38,39,40], and encodes 41 different genes under the control of 5 promoter regions (LEE1–LEE4 and Tir, Figure 2). The T3SS is not constitutively active, and expression is tightly controlled by environmental signaling factors [8,34,36,37,39].

This review will cover a selection of natural product inhibitors of the T3SS, which were chosen to highlight the structural diversity of T3SS inhibitors as well as the diversity of bacteria, plants and fungi that make them. The discovery of natural products as inhibitors of the T3SS introduces new chemical scaffolds for exploration and development of drug-like compounds for clinical investigation, and understanding their mechanisms of action will create opportunity for rational drug design for more potent compounds. 

## 2. Natural Products

### 2.1. Caminosides

**Figure 3:**
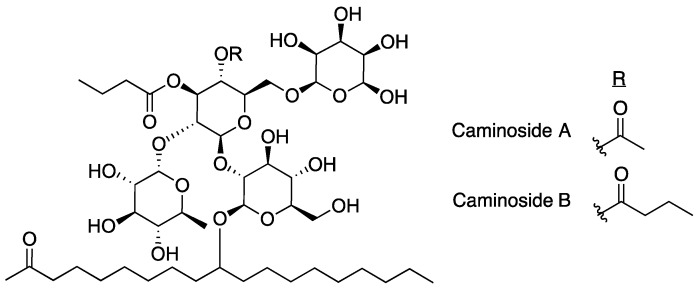
Structures of caminosides, inhibitors isolated from marine sponge *C. sphaeroconia*.

The first inhibitor of the T3SS discovered was caminoside A (Figure 3) [15]. The caminosides are glycolipids isolated from the marine sponge *Caminus sphaeroconia* found in the upper walls of the Toucari Caves on the island of Dominica. Marine invertebrates were collected and transported to the Anderson lab facilities in Canada, where they were extracted repetitively with methanol before screening for T3SS inhibitory activity in EPEC. The isolation of caminoside A was a result of a bioassay guided fractionation approach using a protocol designed to screen for T3SS inhibitors using sodium dodecyl sulfate polyacrylamide gel electrophoresis (SDS-PAGE) and analyzing secretion of effector proteins (Esps). Samples having cytotoxic effects against EPEC were dropped from the study. 

Caminoside A decreased the secretion of EspB, but not EspC. Since EspC is also secreted via the type IV secretion system this result indicates that caminoside A is specifically targeting the T3SS [41]. The structure of caminoside A was elucidated and potency was characterized (IC_50_ = 20 μm), although details on the mechanism of action are still not well understood [15]. Interestingly, although caminoside A has no cytotoxic effect against EPEC, it does have cytotoxic activity against Gram-positive methicillin-resistant *Staphylococcus aureus* (MRSA) and vancomycin-resistant *Enterococcus* (MIC = 12 μg/mL for each). Since the discovery of the caminosides, full syntheses of caminoside A and B have been published [42,43], detailing a synthetic process of 33 and 25 steps, respectively. Despite the promising activity of the caminosides against EPEC, very little has been done since their discovery to further develop these natural products for use as T3SS inhibitors due to difficulties in production or synthesis of the compounds.

### 2.2. Aurodox

**Figure 4:**
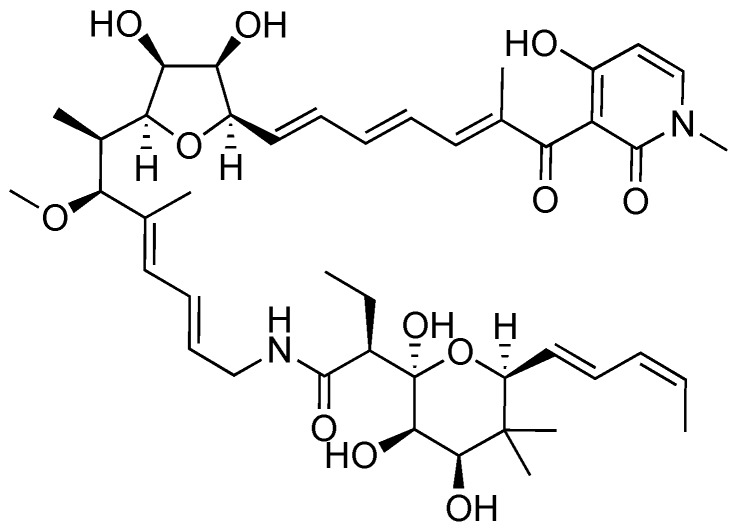
Structure of aurodox, a polyketide isolated from *Streptomyces* sp. K06-0806.

Aurodox, a polyketide produced by *Streptomyces goldiniensis*, was originally isolated and characterized as having antibiotic activity in 1973 (Figure 4) [44]. Aurodox was originally named antibiotic X-5108 by the Grunberg lab when they elucidated its structure and determined aurodox’s antibiotic activity against Gram-positive bacteria. The Chinali lab investigated the biological activity of aurodox by performing structure activity relationship (SAR) studies against the biological target elongation factor Tu (EF-Tu) [45].

Aurodox was identified as a T3SS inhibitor by the Abe group in 2011 after the development of a method to screen for inhibitors of the T3SS in EPEC known as EPEC-mediated hemolysis [14,46]. The molecular components of the translocon, EspB and EspD, typically form the end of the T3SS needle complex and allow for passage of effectors into the target host cell [24,25,26]; however, they also form pores on the surface of red blood cells (RBCs) [46,47]. Formation of these pores results in leakage of hemoglobin into the extracellular space. The supernatant may then be separated from cellular components and the concentration of hemoglobin may be measured indirectly by absorbance measurements. Thus, hemoglobin concentrations can be tied to T3SS expression. 

A screen was performed on 13,300 biological extracts from actinomycetes, fungi, plants, and invertebrates [14]. After extracts from *Streptomyces* sp. K06-0806 showed potent inhibition of EPEC-mediated hemolysis of RBCs without significantly affecting bacterial growth, a large culture of *Streptomyces* sp. K06-0806 was fermented and aurodox was purified. Further testing with purified compound confirmed inhibitory potency of aurodox in the RBC assay (IC_50_ = 1.5 μg/mL). According to analysis by SDS-PAGE followed by Western blotting, aurodox reduces the amount of secreted proteins EspA, EspB, EspD, EspF and Map (an effector that targets and damages host cell mitochondria [48]) without significantly affecting overall protein levels. It was shown that T3SS inhibition (IC_50_ = 1.5 μg/mL) occurs at a concentration much lower than the concentration at which aurodox shows signs of toxicity against Gram-negative bacteria (~10 μg/mL) [14].

The Abe lab collected further data using an *in vivo* mouse model using *Citrobacter rodentium* [40] to analyze the effectiveness of aurodox on mitigating infection. *C. rodentium* is a commonly used model of EPEC infection in mice, due to a high identity of sequence between the EPEC LEE and the LEE in *C. rodentium* [14,40]. Mice were initially infected with *C. rodentium*, then treated either with 10% dimethyl sulfoxide as a control, a single dose of tetracycline (200 mg/kg), or aurodox (25 mg/kg) every 24 hours for four days. All of the mice that were treated with aurodox survived while none of those that were treated with tetracycline survived past day 13. These results show the power of T3SS inhibitors to protect against an otherwise lethal dose of pathogen. 

A recent study has been published investigating the mechanism of action of aurodox [49]. The Roe lab showed that aurodox decreased the secretion of effector proteins via Western blotting. Aurodox was also shown to decrease infectability of epithelial cells by EHEC. Transcriptomal analysis on gene expression revealed that aurodox downregulates more than 100 genes cell-wide and downregulates 25 of 41 genes related to the T3SS. This suggests that the inhibitory activity of aurodox is a result of a change in gene expression, and not a result of physical manipulation of the T3SS needle complex. One of the genes downregulated by aurodox is *ler*, a major activator of the LEE [28]. In addition, aurodox downregulated the expression of EspG and NleB, which are non-LEE encoded effectors. Importantly, treatment with aurodox does not induce Shiga toxin production in EHEC, suggesting promise for the use of aurodox to treat EHEC infection. If the binding target of aurodox were identified, efforts could be made to strengthen that binding and increase the potency of aurodox further.

### 2.3. Piericidin A

**Figure 5:**
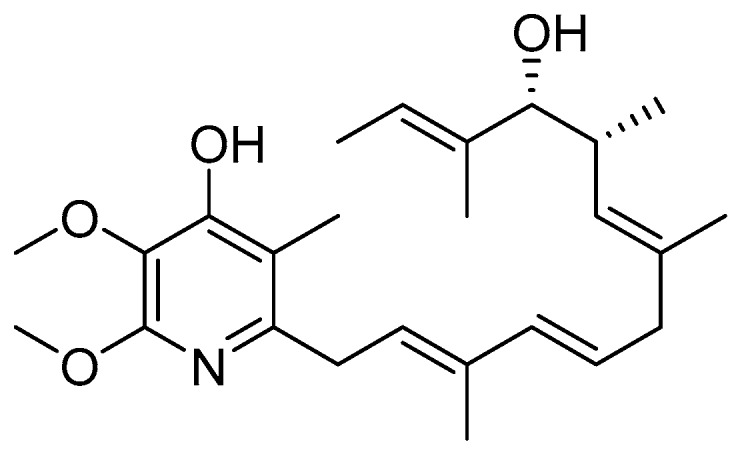
Piericidin A, a natural product T3SS inhibitor isolated from *Streptomyces* sp. 16–22 [50].

Piericidin A was originally discovered for its insecticidal properties in 1963 (Figure 5) [50]. Soil samples were collected from Chiba Prefecture, a region encompassing the outskirts of Tokyo, and their microbial makeup was analyzed for their toxicity against a variety of larval species. The microorganism that exemplified the highest toxicity was *Streptomyces* sp. 16–22. Piericidin A was purified via bioactivity-guided fractionation approaches and its structure and chemical properties were characterized. Notably, this same study found that piericidin A presented limited cytotoxicity against Gram-negative bacteria such as *E. coli* and *Xanthymonas oryzae* [50]. In 1966, piericidin A was investigated for antibiotic properties against Gram-positive bacteria [51]. It was discovered that piericidin A targets nicotinamide adenine dinucleotide (NADH) dehydrogenase within complex I (100% inhibition at 0.03 nmol piericidin per mg protein), a complex important in mitochondrial electron transport. In 2010, the Svatos lab described a symbiotic relationship between beewolf digger wasps and certain strains of piericidin-producing *Streptomyces* [52]. The wasps cultivate *Streptomyces* on their antennae, and incorporate the cells into their larval cocoons as prophylaxis against pathogenic bacteria. This is just one example of nature’s purposeful use of natural products as a defensive mechanism against pathogens.

In 2014, the Auerbach lab performed a high-throughput screen to discover new inhibitors of the *Yersinia pseudotuberculosis* T3SS and uncovered piericidin A’s inhibitory activity [53]. *Y. pseudotuberculosis* possesses a unique ability to trigger Nf-κB signaling in HEK293T cells, a process that is dependent on YopB and YopD transport of effectors into the host cytosol [54]. T3SS function was measured using an Nf-κB luciferase reporter plasmid and changes in activity were monitored to identify potent inhibitors [53]. After eliminating compounds that produced cytotoxicity against either the mammalian or bacterial cells, the group identified piericidin A as one of their hit compounds. SDS-PAGE analysis indicated that secretion of YopE was decreased by 65% at 71 μm piericidin A. Piericidin A was also shown to potently inhibit translocation of YopM (75% decrease at 71 μm) into Chinese hamster ovary (CHO) cells.

A clue into the mechanism of action of piericidin A as a T3SS inhibitor has recently been discovered. Inhibition of the T3SS by piericidin A resulted in decreased formation of Ysc-type needle units on the surface of *Y. pseudotuberculosis* without interfering with gene expression, indicating the mechanism of action is related directly to needle assembly [54]. Although piericidin has a known antibacterial target (Complex I), an alternative Complex I inhibitor, rotenone, has no T3SS inhibitory activity, indicating the T3SS inhibitory activity of piericidin A may be independent of complex I inhibition [55]. Further work to find the binding partner to elicit T3SS inhibition by piericidin A would aid in the ability to rationally design more potent analogs that selectively inhibit the T3SS without producing antibiotic effects related to Complex I binding.

### 2.4. Cytosporone B

**Figure 6:**
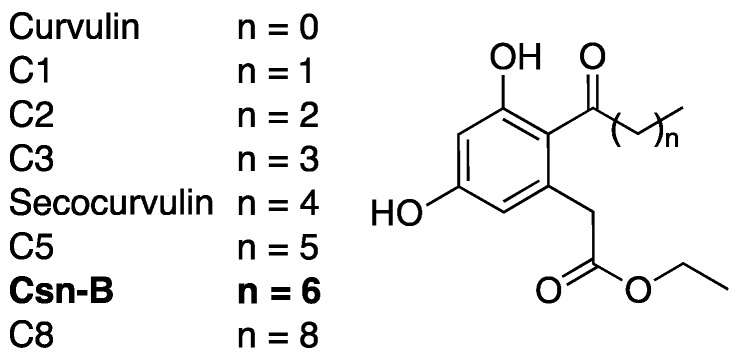
Analogs of cytosporone B (Csn-B) synthesized and analyzed for T3SS inhibitory activity by the Shen group.

Cytosporone B (Csn-B) was originally identified as a naturally occurring substrate to nuclear orphan receptor Nur77 in 2008 [56]. This octaketide natural product (Figure 6), isolated from the endophytic fungus *Dothiorella* sp. HTF3, has since been extensively studied as a potential anti-cancer agent due to its ability to stimulate Nur77-mediated apoptosis in multiple cancer cell models [56,57,58]. Csn-B was later identified by the Shen lab as an inhibitor of the T3SS in *Salmonella enterica* serovar Typhimurium during a screen of Csn-B and analogues [59].

Western blots indicated that secocurvulin, C5, Csn-B, and C8 (Figure 6) were all capable of inhibiting secretion of SPI-1 effectors in *S. enterica* [60]. In addition, secocurvulin, C5, and Csn-B inhibited *S. enterica* invasion of HeLa cells, a process dependent on SPI-1, with Csn-B proving to be the most potent. General SAR analysis suggests that the inhibitory potency is maximized at *n* = 6 (Csn-B), and that the potency decreases with increased or decreased chain length. Csn-B also showed dose-dependent inhibition of SPI-1 effector secretion (IC_50_ = 6.25 μm) with no toxicity to bacterial cells. Although the molecular target for T3SS inhibition is unknown, Csn-B inhibition could be overcome by overexpression of HilA, a positive regulator of the *S. enterica* T3SS. This result suggests that Csn-B interferes with the HilA expression pathway. A route for Csn-B total synthesis was published in 2010 [61], but since then, very little has been done to further this compound as a T3SS inhibitor.

### 2.5. Guadinomines

**Figure 7:**
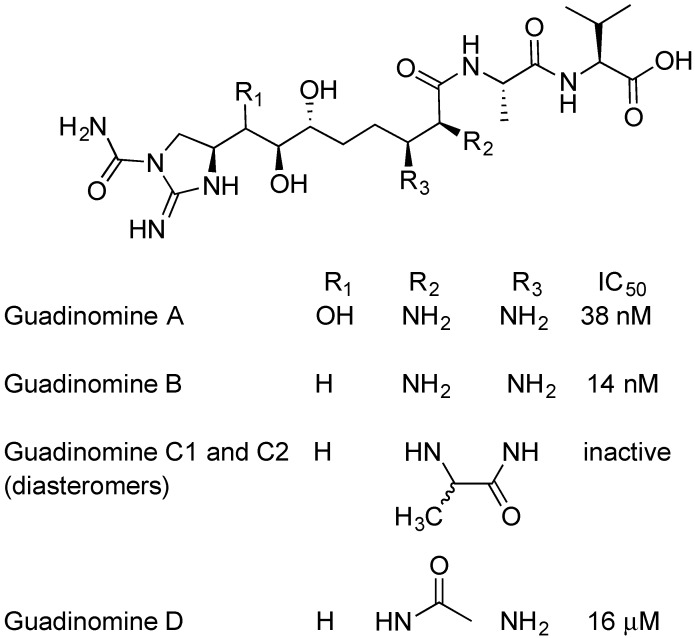
Guadinomines isolated from *Streptomyces* sp. K01-0509, and their activity against enteropathogenic *Escherichia coli* (EPEC).

The guadinomines were discovered by Ōmura and colleagues using EPEC-mediated hemolysis to screen natural product extracts. [62,63]. *Streptomyces* sp. K01-0509 was found to produce potent inhibitors of RBC hemolysis [45,46]. Cultures were scaled up, and guadinomine A, B, C1, C2, and D were isolated, purified and analyzed (Figure 7) [62]. Guadinomines A and B are the most potent natural product TTSS inhibitors with IC_50_ = 0.007~0.01 μg/mL. The mechanism of action of the guadinomines is not well understood and yields of guadinomines from culture are low, making further research difficult. The lengthy total syntheses of guadinomine B and C2 have been published, with 33 steps in the longest linear synthetic sequence [64]. In 2012, a study on the biosynthetic pathway of guadinomine A was published by Khosla and coworkers [65]. Notably, guadinomine D, having an acylated amine at R_2_ (Figure 7), is 1000-fold less potent than guadinomine B. This shows the importance of the vicinal diamine to biological activity. The acyl group on guadinomine D may be installed by enzymes apart from the guadinomine synthetase, since no obvious acylation enzyme is part of the gene cluster. While guadinomines do not appear to produce any antimicrobial activity, Ōmura found that guadinomine B is cytotoxic to Jukat cells at a concentration 100 times higher than the IC_50_ for TTSS inhibition [62].

### 2.6. Butyric Acid

**Figure 8:**
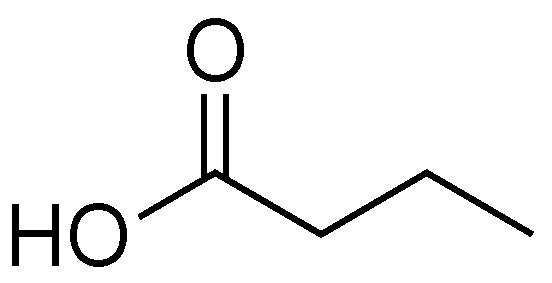
Butyric acid, a product of Bacterioidetes and other intestinal microbes [67].

Initial studies relating to the biological effects of butyric acid predate knowledge of the T3SS (Figure 8). In the 1960s, William R. Martin and coworkers investigated how the infectious dose (ID_50_) of *Salmonella enteritidis* changed when mice were pretreated with antibiotics [66]. The authors showed that the ED_50_ went from 10^6^ to <10 cells when a single dose of streptomycin was administered 24 hours before infection. The authors noted that treatment with antibiotic increased the gut pH and identified that butyric acid and acetic acid were being produced by the gut bacteria. The authors attributed the ability of the mice to tolerate *Salmonella* to a low gut pH and not a specific inhibitory effect caused by butyric acid. Since these experiments were performed before the discovery of the T3SS, the authors were unaware that they were completing initial studies on the effect that T3SS inhibitors have on severity of infection. 

Butyric acid is classified as a short-chain fatty acid (SCFA), and is produced as a fermentation product by commensal Bacterioidetes species in mammals. In human intestines butyric acid is typically present at 10–20 mm [67,68,69]. Butyric acid is a major energy source for colonocytes, and the ability of colonic cells to absorb and utilize sodium butyrate is seen as a sign of good health [69]. Administration of butyric acid to the intestines of mice infected with *C. rodentium* results in decreased inflammation and increased mucus production from colonic cells.

Butyric acid interacts with the epigenetic modifier Lrp, a major regulator of gene expression in bacteria [5,66,67,68,69]. Lrp does not control the expression of genes in the same pattern from one organism to another. As a result, butyrate acts as a T3SS inhibitor for some organisms and as a T3SS activator in others [68,70,71,72]. A notable example involves LEE-encoding bacteria EPEC and *C. rodentium*. EPEC and *C. rodentium* have 90% sequence identity in their LEE pathogenicity islands [40]. Their T3SSs are so similar that *C. rodentium* is often used as a mouse model for EPEC infection [14]. Lrp is a non-LEE encoded T3SS regulator, and activation of Lrp has opposite responses in these two organisms. Activation of Lrp upregulates expression of the LEE in EPEC, while activation of Lrp downregulates expression of the LEE in *C. rodentium* [68,70,72]. 

Research into SCFAs as T3SS regulators has focused primarily on the effects of probiotics on infection [67,70,71]. By increasing the concentration of SCFA-producing bacteria in the gut, concentrations of a variety of SCFAs are altered. Depending on the pathogen attempting to infect the gut, differing ratios of SCFAs could have dramatically different results, from improving to worsening infection. Given the prevalence and widespread use of probiotics, this area requires further investigation.

### 2.7. Fusaric Acid

**Figure 9:**
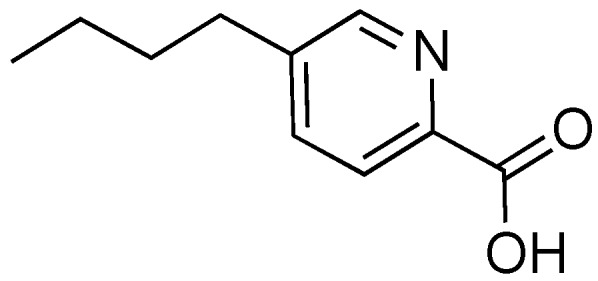
Fusaric acid, a fungal toxin isolated from *Fusarium oxysporum.*

Fusaric acid is a toxin produced by fungal species *Fusarium oxysporum*, a common inhabitant of soil (Figure 9) [73,74]. Fusaric acid causes a variety of negative outcomes in plants, and it is thought to be a virulence factor in Fusarium wilt in banana, tomato, and cotton crops [73,74,75] and heavy decline disease in grapevine [76]. Fusaric acid was first studied as a potential inhibitor of the T3SS in *S. enterica* in 2014 as part of a screen of a small library [77]. In SDS-PAGE and Western blot analysis, fusaric acid potently inhibited secretion of SPI-1 effector proteins when cells were treated with 100 μm fusaric acid, without disrupting cell growth. Inhibition was dose-dependent with a calculated IC_50_ = 53.5 μm. In a gentamycin-protection assay, fusaric acid markedly inhibited *Salmonella* invasion into HeLa cells, with no toxicity toward the HeLa cells observed [77]. 

Some studies attempting to elucidate the mechanism of action of fusaric acid gave conclusive results that the inhibitory effect of fusaric acid cannot be overcome by overexpression of T3SS activator HilA, unlike the case of Csn-B [77]. Also, fusaric acid does not change the level of PgrH, an assembly protein for the needle complex, and does not interfere with the SicA/InfV transcriptional pathway for T3SS initiation. Further studies on the mechanism of action are needed in order to determine the pathway through which fusaric acid elicits an inhibitory response. The cytotoxicity of fusaric acid against plants and other organisms will need to be considered when moving forward with this compound as a potential T3SS inhibitor.

### 2.8. (-)-Hopeaphenol

**Figure 10:**
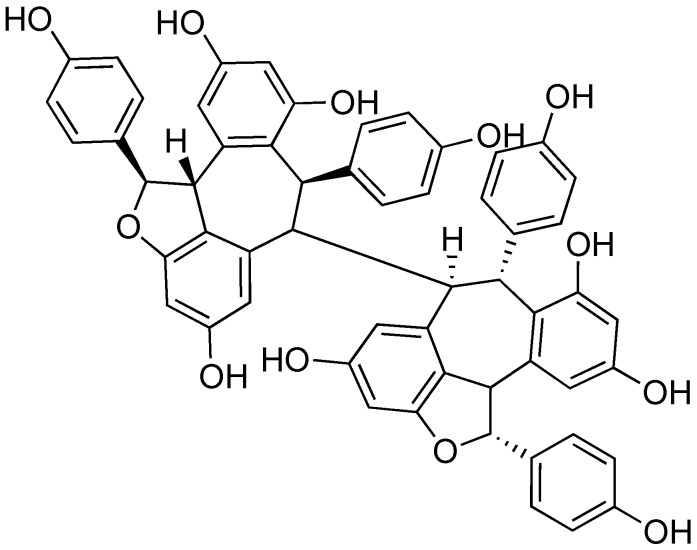
(-)-Hopeaphenol, a tetramer of resveratrol isolated from rainforest plants.

Many plants produce inhibitors to protect against infection caused by Gram-negative pathogens that utilize a T3SS. (-)-Hopeaphenol (Figure 10) was isolated as part of a bioassay-guided fractionation study to find natural product T3SS inhibitors from two rainforest plants from Papua New Guinea, *Anisoptera thurifera* and *A. polyandra* [78]. (-)-Hopeaphenol is a tetramer of resveratrol, a common building block used in nature to synthesize natural products. (-)-Hopeaphenol has also been explored for its anti-oxidant properties [79]. In their work, Eloffson and coworkers analyzed (-)-hopeaphenol for inhibition of T3SS in *Y. pseudotuberculosis*, *P. aeruginosa*, and *C. trachomatis* [78]. (-)-Hopeaphenol exhibited inhibition of YopE expression in a reporter gene assay and found the compound had inhibitory activity (IC_50_ = 6.6 μm). Western blot analysis showed dose-dependent inhibition of secretion and expression of YopD. When cells grown in the presence of (-)-hopeaphenol were moved to T3SS-inducing environments, they were incapable of expressing the T3SS, regardless of whether (-)-hopeaphenol was still present. This suggests an irreversible mechanism of inhibition. 

(-)-Hopeaphenol was also found to inhibit expression and secretion of ExoS, an effector from the *P. aeruginosa* T3SS [79]. In infection model assays, (-)-hopeaphenol completely inhibited infection of HeLa cells by *P. aeruginosa* at a concentration of 100 μm. In addition, (-)-hopeaphenol was observed to inhibit intracellular growth of *C. trachomatis* in HeLa cells in a dose-dependent manner. When tested for cytotoxicity against a panel of Gram-positive and Gram-negative organisms, (-)-hopeaphenol did not affect cell growth or viability. Despite the promise of this structural class, there are large barriers for chemical synthesis. In addition, *Anisoptera* spp. that produce (-)-hopeaphenol are in danger of extinction; 6 of 10 within the genus are either endangered or critically endangered according to the International Union for Conservation of Nature (IUCN) Red List, with the remainder being vulnerable [80]. Without the ability to easily access samples of (-)-hopeaphenol and analogs to test for T3SS inhibitory activity, further development of this structural class as inhibitors will be difficult.

### 2.9. Sanguinarine Chloride

**Figure 11:**
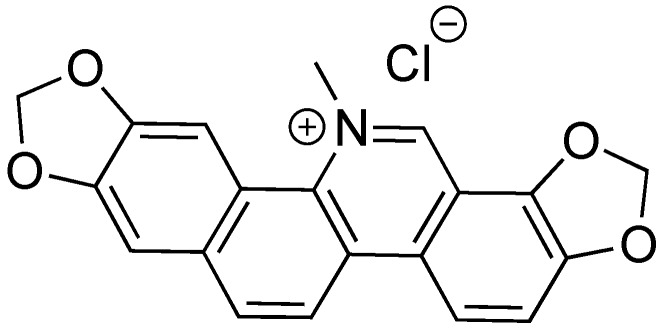
Sanguinarine chloride, a natural product from bloodroot *Sanguinaria canadensis*.

Sanguinarine chloride is a natural product isolated from the extracts of the bloodroot plant *Sanguinaria canadensis* (Figure 11) [81]. In the 1970s and 1980s, sanguinarine chloride was studied as a potential treatment for gingivitis due to its anti-inflammatory properties. It has since been studied as a chemotherapeutic agent [82]. Sanguinarine chloride was found to be a T3SS inhibitor against *Salmonella enterica* serovar Typhimurium [83]. It inhibited SipA-lactamase fusion translocation into HeLa cells at a concentration of 4 μm. In a gentamycin-protection assay, sanguinarine chloride was effective against pathogenic invasion of HeLa cells. Expression of SipA and SipB was also inhibited at 5 μm sanguinarine chloride. Overexpression of HilA overcomes the inhibitory effects of sanguinarine chloride, indicating a possible mechanism of action. While sanguinarine chloride shows promise as a T3SS inhibitor, efforts to reduce the cytotoxic affects that the compound has toward human cells must be made for this drug to move forward, as well as further characterizing its biological target. 

### 2.10. Thymol

**Figure 12:**
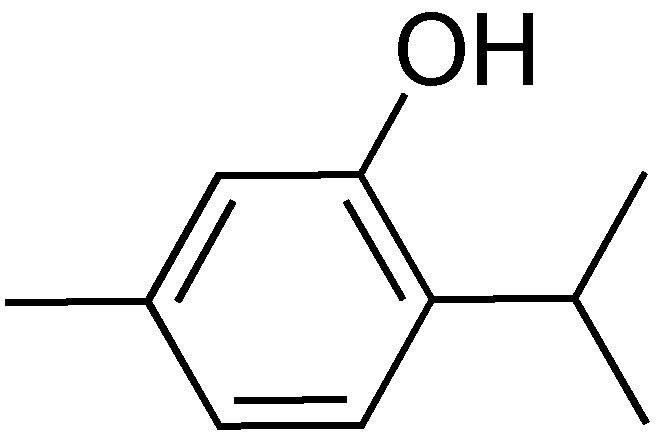
Thymol, a major component of essential oils from *Thymus* plants.

The Deng lab recently identified thymol during a study aimed at identifying T3SS inhibitors from traditional Chinese medicine (Figure 12) [84]. Thymol is a component of an essential oil derived from plants belonging to the *Thymus* genus [85]. The translocation of a SipA-lactamase fusion from *Salmonella* into HeLa cells in the presence or absence of thymol was monitored. At a concentration of 0.2 mm thymol, translocation was almost completely inhibited, while cytotoxicity was not observed until thymol concentrations reached 0.6 mm. In a gentamycin protection assay, preincubation with 0.2 mm thymol resulted in a 90% reduction of T3SS-dependent internalization of *Salmonella* by HeLa cells. Doses of 50 mg/kg thymol resulted in a 100% survival rate of mice administered lethal doses of *Salmonella* after a 10-day infection period. This dose also alleviated pathophysiology related to *Salmonella* infections to colonocytes. These promising results show that further investigations are needed into traditional medicines.

### 2.11. Cinnamic Acid and Derivatives

**Figure 13:**
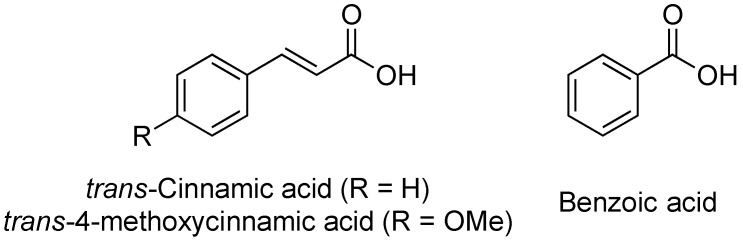
Structures of *trans*-cinnamic acid (TCA), *trans*-4-methoxycinnamic acid (TMCA), and benzoic acid (BA), regulators of the T3SS.

In 2008, the Yang lab investigated the influence of plant-derived compounds on the expression of the T3SS in *Dickeya dadantii* 3937 [86]. They chose to monitor change in expression of the T3SS via the two major regulatory pathways in *D. dadantii* 3937, the HrpX/HrpY-HrpS-HrpL and GacS/GacA-rsmB-RsmA pathways. The group found that *trans*-cinnamic acid (TCA, Figure 13) acts as an activator of the *D. dadantii* T3SS, increasing the expression of the gene *hrpN*, an indicator for the HrpX/HrpY-HrpS-HrpL pathway. Specifically, a 3-fold increase in expression of *hrpN* was observed at 5 μm TCA.

In later studies, the Yang lab investigated derivatives of TCA, including *trans*-4-methoxycinnamic acid (TMCA) and benzoic acid (BA, Figure 13) on T3SS expression in *Erwinia amylovora*, the fire blight pathogen [87]. Their results indicated that TMCA and BA act as inhibitors of T3SS expression in a fused-green fluorescence protein (GFP)-*hrp* reporter assay. TCA, TMCA and BA (100 μm) all decreased fluorescence to approximately 30%, 20%, and 3% of the control, respectively. Based on this assay, the IC_50_ concentrations for TCA (0.5 μm) and BA (1 μm) were approximated. Northern blot analysis indicated that TMCA inhibits the T3SS by reducing expression of *rsmB_Ea_* and *hrpN*, while BA only inhibits the expression of *hrpN*, suggesting potential differences in their mechanisms of action. None of the compounds altered the expression of the regulatory genes *hrpX/hrpY*. Further analyses of this structural class, their mechanisms of action, and the differences in inhibitory/inducing behavior between pathogenic species will need to be conducted for this group of compounds to move forward as T3SS inhibitors.

## 3. Conclusions

Many natural products have been shown to possess T3SS inhibitory properties over the last decade. These compounds are made by a variety of different biological sources and cover a diverse range of chemical scaffolds. Notwithstanding these successes, most inhibitors of the T3SS have unknown biological targets. This complicates the rational design of new and more potent analogs. With a better understanding of their binding partners and mechanism of action, modern methods of analog design (e.g., computational modeling) can be employed effectively. More natural product T3SS inhibitors are still being discovered, indicating that there remains a lot to learn about how nature employs this strategy.
